# The two neutrophil members of the formylpeptide receptor family activate the NADPH-oxidase through signals that differ in sensitivity to a gelsolin derived phosphoinositide-binding peptide

**DOI:** 10.1186/1471-2121-5-50

**Published:** 2004-12-29

**Authors:** Huamei Fu, Lena Björkman, Paul Janmey, Anna Karlsson, Jennie Karlsson, Charlotta Movitz, Claes Dahlgren

**Affiliations:** 1 Department of Rheumatology and Inflammation Research, University of Göteborg, Sweden; 2 Department of Physiology, Institute for Medicine and Engineering, University of Pennsylvania, Philadelphia, PA 19063 USA; 3 Department of Virology, University of Göteborg, Sweden

## Abstract

**Background:**

The formylpeptide receptor family members FPR and FPRL1, expressed in myeloid phagocytes, belong to the G-protein coupled seven transmembrane receptor family (GPCRs). They share a high degree of sequence similarity, particularly in the cytoplasmic domains involved in intracellular signaling. The established model of cell activation through GPCRs states that the receptors isomerize from an inactive to an active state upon ligand binding, and this receptor transformation subsequently activates the signal transducing G-protein. Accordingly, the activation of human neutrophil FPR and FPRL1 induces identical, pertussis toxin-sensitive functional responses and a transient increase in intracellular calcium is followed by a secretory response leading to mobilization of receptors from intracellular stores, as well as a release of reactive oxygen metabolites.

**Results:**

We report that a cell permeable ten amino acid peptide (PBP10) derived from the phosphatidylinositol 4,5-bisphosphate (PIP_2_) binding region of gelsolin (an uncapper of actin filaments) blocks granule mobilization as well as secretion of oxygen radicals. The inhibitory effect of PBP10 is, however, receptor specific and affects the FPRL1-, but not the FPR-, induced cellular response. The transient rise in intracellular calcium induced by the active receptors is not affected by PBP10, suggesting that the blockage occurs in a parallel, novel signaling pathway used by FPRL1 to induce oxygen radical production and secretion. Also the FPR can activate neutrophils through a PBP10-sensitive signaling pathway, but this signal is normally blocked by the cytoskeleton.

**Conclusions:**

This study demonstrates that the two very closely related chemoattractant receptors, FPR and FPRL1, use distinct signaling pathways in activation of human neutrophils. The PIP_2_-binding peptide PBP10 selectively inhibits FPRL1-mediated superoxide production and granule mobilization. Furthermore, the activity of this novel PBP10 sensitive pathway in neutrophils is modulated by the actin cytoskeleton network.

## Background

The molecular basis for cellular recognition of signal molecules is their binding to specific cell surface receptors [1]. Despite large structural differences between the huge numbers of extracellular ligands, many bind to (and activate) specific receptors belonging to a large family of pertussis toxin-sensitive G-protein linked receptors (GPCRs) [2]. These receptors possess a high degree of similarity and although activated by different agonists, they transduce downstream signals that have many common features [3]. Nevertheless, it is clear that there are also important differences between receptor-ligand pairs regarding their functional repertoire [4]. The pattern recognition, formyl peptide receptor (FPR) family, belongs to the larger GPCR group of chemoattractant receptors [5]. The FPR gene family has a complex evolutionary history and the number, function, and specific cell expression of genes comprising the family vary considerably between different mammalian species [5,6]. Human neutrophil granulocytes express two FPR members [7], the FPR and the FPRL1 (the formyl peptide receptor like 1). FPRL1 was originally defined as an orphan receptor, cloned from an HL-60 cell cDNA library by low-stringency hybridization with the FPR sequence [7]. In the past few years several neutrophil activating ligands specific for FPRL1 have been identified [8], but knowledge on the precise functional activities and the signal transduction pathways utilized by FPRL1 are still somewhat limited. In contrast to FPRL1, a large number of studies on FPR induced cell function and signaling have been performed in neutrophils and in receptor-expressing transfected cell lines. These studies reveal that FPR signaling shows all the basic characteristic of a GPCR. Binding of the prototype FPR agonist fMLF to its receptor initiates a chain of events starting with dissociation of the G-protein α subunit from its βγ subunit. These subunits directly or indirectly activate downstream signaling molecules such as protein kinase C (PKC), mitogen-activated protein kinase (MAPK), and phosphoinositide 3-kinase (PI3K) that uses the membrane phosphoinositide phosphatidylinositol 4,5-bisphosphate (PIP_2_) as substrate [9]. The dissociated subunits of the G-protein also activate the phosphoinositide-specific phospholipase C (PLC) that upon cleavage of PIP_2 _produces the second messengers responsible for an elevation of intracellular free calcium [8]. It has been assumed that FPRL1 shares signal transduction features with FPR, since both receptors are sensitive to pertussis toxin and possess a high degree of amino acid identity in the signaling cytoplasmic domains [7]. Further, the functional responses induced by the FPRL1 specific hexapeptide agonist WKYMVM is in most respects similar to (or even indistinguishable from) those induced by the prototype FPR agonist fMLF [10-15]. However, in this study we have used a membrane permeant polyphosphoinositide-binding peptide (PBP10 [16]) derived from the cytoskeletal protein gelsolin, and we show that with respect to messengers generated by the these receptors leading to mobilization of secretory granules and NADPH-oxidase activation, two totally different signaling routes are used by FPR and FPRL1, one being sensitive (the FPRL1 route) the other insensitive (the FRP route) to the PIP_2_-binding peptide.

## Results

### NADPH-oxidase activity induced by fMLF and WKYMVM and effects of PBP10

**Figure 1:**
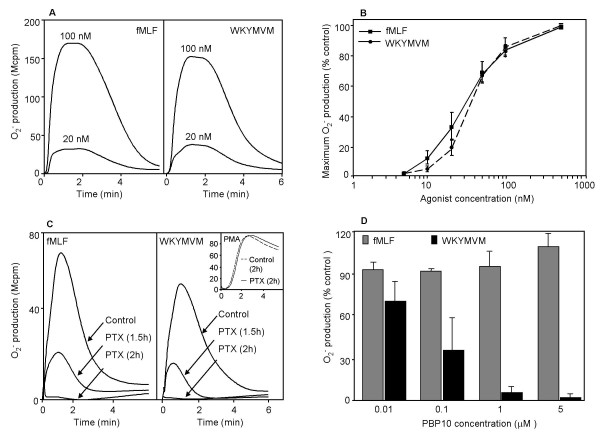
**Oxidase activity induced by peptide agonists and the effect of PBP 10. **Neutrophils were activated by fMLF or WKYMVM and the extracellular release of superoxide anions was recorded by chemiluminescence (expressed in Mcpm). (A) The figure shows the kinetics of the neutrophil response to two concentrations (100 nM and 20 nM) of fMLF or WKYMVM. (B) Dose dependent oxidase activation induced by the two agonists. The peak values were measured and the responses are given as percent of the maximal response. (C) Neutrophils were incubated for 120 minutes in the absence (control) or presence of pertussis toxin (PTX; 500 ng/ml) and the cells were then activated with fMLF (100 nM), WKYMVM (100 nM) or the receptor independent PKC activator PMA (100 nM) (inset). For comparision, the response induced by the two peptides after 90 minutes long incubation time with pertussis toxin is also included. (D) Effect of different concentrations of PBP10 on the neutrophil NADPH-oxidase response induced by fMLF or WYMVM, respectively. Data are expressed as percent of control (without PBP10; mean ± SD of three independent experiments).

The peptides fMLF and WKYMVM, agonists for FPR and FPRL1, respectively, both induced a robust oxidative burst measured as a release of superoxide anions (Fig 1). In accordance with the known receptor specificity [10,17], the WKYMVM response was totally inhibited by the FPRL1 specific antagonist WRWWWW whereas the FPR specific antagonist (cyclosporine H)was without effect. The effects were reversed for fMLF-triggered activity, that is, this response was totally inhibited by cyclosporine H but not affected by WKWWWW (data not shown). The time-courses of the responses were very similar, as were the EC_50 _values (Fig. 1A,B). Both the FPR and FPRL1 mediated response was inhibited by pertussis toxin (whereas no reduction in oxidase activity was seen with PMA, a PKC activator that bypasses the G-protein), showing that a heterotrimeric G-protein is involved in the signal transduction of both receptors (Fig. 1C). These indistinguishable responses were expected, based on the fact that the two receptors are very similar in the regions suggested to be of importance for intracellular signaling.

Despite the indistinguishable activation by FPR and FPRL1 shown in Fig. 1A–C, functional differences between these two highly homologous receptors emerge when they are treated with the membrane permeant polyphosphoinositide-binding peptide rhodamine-B-QRLFQVKGRR (PBP10; Fig. 1D) prior to activation. The neutrophil NADPH-oxidase activity was totally inhibited by PBP10 when FPRL1 was stimulated by WKYMVM, whereas there was no effect of the peptide on the fMLF-induced, FPR-mediated neutrophil response. The IC_50 _value for the WKYMVM induced response was around 0.05 μM whereas no effect was seen on the fMLF induced activity even at concentrations up to 10 μM. There was no effect on WKYMVM or fMLF induced superoxide production of rhodamine alone (data not shown).

### The neutrophil response is inhibited by Wortmannin

**Figure 2:**
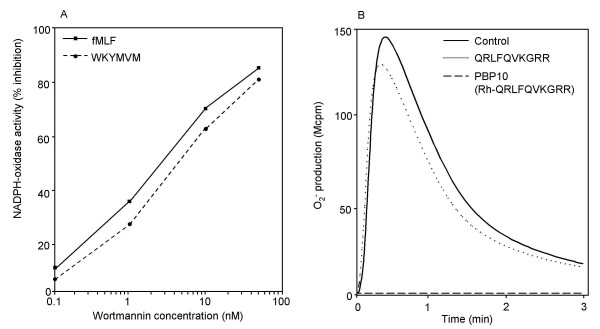
**Effect of different inhibitors on the neutrophil NADPH-oxidase activity. **(A) Neutrophils were incubated with different concentrations of the PI3K specific inhibitor Wortmannin at 37°C for 30 minutes followed by stimulation with fMLF (100 nM) or WKYMVM (100 nM). A representative experiment is shown. (B) Neutrophils were incubated with the cell impermeable PIP_2 _binding peptide QRLFQVKGRR (1 μM final concentration) at 37°C for 5 minutes followed by stimulation with WKYMVM (100 nM). The effect of PBP10 (1 μM) is included for comparison.

The amino acid sequence of PBP 10 peptide corresponds to the phosphatidylinositol 4,5-bisphosphate (PIP_2_) binding region segment 2 of the cytoskeletal protein gelsolin [16]. Following G-protein coupled receptor (GPCR) activation, the dissociated G-protein subunits activate the downstream phosphoinositide remodeling enzyme phosphatidylinositol 3-kinase (PI3K) [18], an enzyme that uses the membrane phosphoinositide PIP_2 _as substrate to generate the signaling molecule phosphatidylinositol 3,4,5-trisphosphate (PIP_3_) [9]. The oxidase activity induced by fMLF as well as by WKYMVM was largely inhibited by the specific PI3K inhibitor Wortmannin (Fig. 2A) suggesting that this signaling pathway is of importance in the cellular response. In contrast to the effect of PBP10, the effects of the PI3K inhibitor showed no specificity in the inhibition of the WKYMVM/FPRL1 triggered response.

### PBP 10 inhibits the response to FPRL1 but not to FPR

The inhibition induced by PBP10 on neutrophil oxidase activity was linked to the receptor FPRL1 rather than to an FPRL1-specific agonist, since the same inhibition pattern was obtained when WKYMVM was replaced by serum amyloid A (SAA) (Table 1), an agonist that activates cells through binding to FPRL1 [19]. Moreover, no effect was induced by PBP10 when fMLF was replaced by annexinI_9–25 _(Table 1), a 17 amino acid peptide derived from the N-terminus of annexin I that has been shown to be a FPR agonist ([20] and our own observation). Moreover, the cellular responses mediated through other receptors, such as the chemokine receptor CXC or the C5a-receptor were not affected by PBP10 (Table 1).

**Table 1:** Effect of PBP10 on neutrophil NADPH-oxidase activity induced by receptor agonists^#^.

Neutrophil NADPH-oxidase activator (Concentration)	Neutrophil receptor(s)	Inhibition of NADPH-oxidase by PBP10 (1 μM)
WKYMVM (100 nM)	FPRL1	+
SAA (5 μM)	FPRL1	+
fMLF (100 nM)	FPR	-
Annexin_9–25 _(50 μM)	FPR	-
C5a (100 ng/ml)	C5aR	-
IL-8 (100 ng/ml)	CXCR1, CXCR2	-

Abbreviations: C5a, complement 5a; FPR, formyl peptide receptor; IL-8, interleukin 8; SAA, serum amyloid A.

### The membrane permeability of PBP10 is necessary for FPRL1 inhibition

Interaction of PBP10 with PIP_2 _depends on the peptide sequence, as truncation of the peptide either at the N terminus or C terminus reduces the PIP_2 _binding affinity [21]. Accordingly, Rh-VKGRRG affected the oxidase activity induced by WKYMVM only when higher concentrations of peptide were used (IC_50 _= 1 μM compared to 0.05 μM for PBP10) suggesting that PIP_2 _binding is of importance for inhibition of the neutrophil oxidase response induced by FPRL1.

The PBP10 peptide possesses not only PIP_2 _binding capacity but is also membrane permeable. This feature has been shown to be coupled to the rhodamine B part of the molecule, i.e., the unconjugated peptide QRLFQVKGRR exhibits the same PIP_2_-binding characteristics as PBP10 but lacks the ability to cross the plasma membrane [16]. The unconjugated peptide had no effect on the WKYMVM induced neutrophil oxidase activity (Fig. 2B) suggesting that membrane permeation is required for peptide-induced inhibition.

### No effect of PBP10 on the transient calcium response

**Figure 3:**
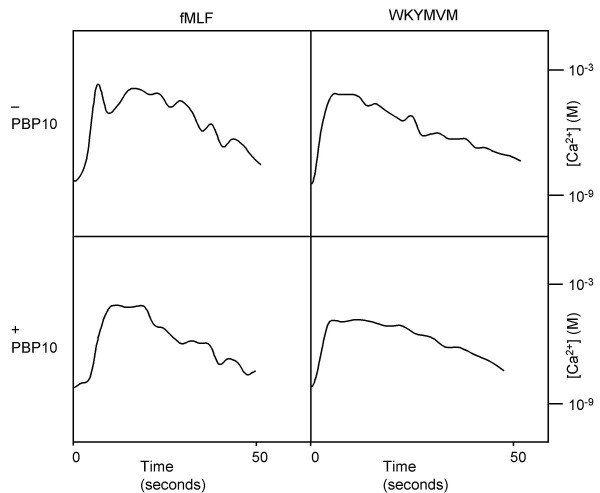
**Effect of PBP10 on cytosolic calcium mobilization induced by peptide agonists. **Neutrophil intracellular calcium mobilization was analyzed by monitoring Fura-2 fluorescence upon stimulation with the FPR agonist fMLF (100 nM; left) or the FPRL1 agonist WKYMVM (100 nM; right) in the absence (upper curves) or presence (lower curves) of PBP10 (1 μM). The curves are derived from a representative experiment. The calcium concentrations given in the right hand part of the figure are valid for the fMLF as well as the WKYMVM induced responses.

PIP_2 _is utilized for generation of IP_3 _that induces a transient increase in intracellular calcium [22], we thus investigated the effect of PBP10 on the neutrophil calcium response. Stimulating the cells with fMLF or WKYMVM induced a rapid and transient increase in cytosolic calcium. These responses were not significantly reduced by the removal of extracellular Ca^2+ ^with EGTA (data not shown), suggesting that the rise in intracellular free Ca^2+ ^relates to a mobilization from calcium storing organelles rather then from an influx through ion channels in the plasma membrane. Treatment of the cells with PBP10 prior to stimulation did not alter the calcium responses, neither induced by fMLF nor by WKYMVM (Fig. 3). Hence, the PBP10-induced effect on FPRL1 signaling is not due to a specific inhibition of the calcium transients induced by the activated GPCRs.

### PBP10 inhibits mobilization of CR3

**Figure 4:**
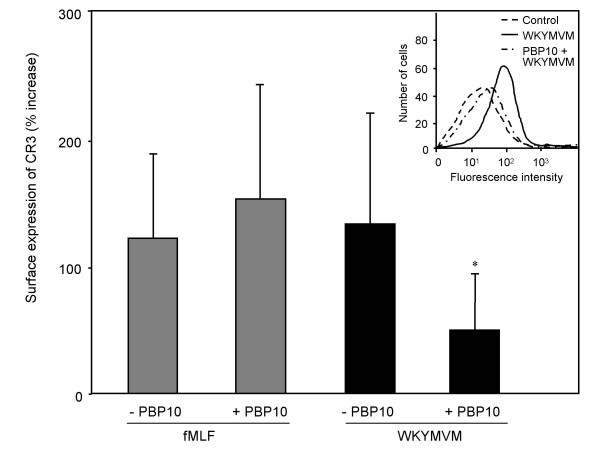
**Effect of PBP10 on fMLF and WKYMVM induced mobilization of CR3 to the neutrophil cell surface. **Neutrophils were incubated with or without PBP10 (1 μM) at 37°C for 5 minutes after which the cells were challenged with fMLF (100 nM) or WKYMVM (100 nM) for another 10 minutes. After fixation of the cells with paraformaldehyde and labeling with anti-CR3 antibody, the amount of CR3 on the cell surface was determined by FACS analysis. A representative histogram of PBP10 effect on WKYMVM mediated CR3 exposure is shown (inset), and the exposure of CR3 after cell stimulation with fMLF and WKYMVM is expressed as percent of control (without PBP10; mean ± SD of four independent experiments). *p < 0.05 compared to WKYMVM without PBP10.

The Ca^2+ ^elevation has been claimed to be required but not sufficient for the generation of an NADPH-oxidase activating signal from FPR [23,24]. Likewise, the mobilization through regulated exocytosis of neutrophil secretory storage organelles containing reserve pools of cell-surface receptors [25] has been suggested to be directly regulated by the cytosolic concentration of free Ca^2+ ^[26,27]. We found that despite the fact that PBP10 was without effect on the transient rise in intracellular Ca^2+ ^induced by WKYMVM, it blocked the secretory response (Fig. 4). In accordance with the inhibition of the NADPH-oxidase activity, PBP10 was without effect on the FPR induced granule mobilization (Fig. 4). This suggests that degranulation and NADPH-oxidase activity induced by FPRL1 both are on the same, PBP10-sensitive, signal transduction pathway in contrast to FPR, which induces both cellular functions by a PBP10-insensitive pathway.

### Regulation by the cytoskeleton

Regulation of FPR signaling has been suggested to involve mechanisms that depend on direct receptor interaction with the membrane cytoskeleton [28]. It is well known that the neutrophil response to FPR and FPRL1 agonists is both augmented and prolonged in the presence of cytochalasin B [15], a fungal metabolite that inhibits re-organization of actin polymers and uncouples receptors from the cytoskeleton [13].

**Figure 5:**
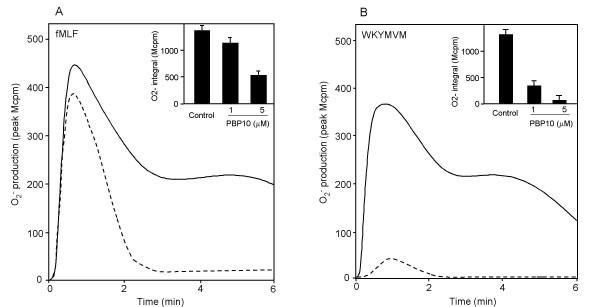
**Effect of PBP10 on oxidase activity induced by peptide agonists in the presence of cytochalasin B. **Neutrophils were activated by fMLF (100 nM) or WKYMVM (100 nM) in the presence of cytochalasin B (2.5 μg/ml, final concentration) and the extracellular release of superoxide anion was recorded (expressed in Mcpm). (A) The figure shows the kinetics of the neutrophil response to fMLF in the absence (solid line) and presence (broken line) of PBP10 (5 μM). The results obtained are summarized in the inset, expressed as the integral values of oxidase activities from the controls (presence of cytochalasin B but not PBP10) and the activities in the presence of both cytocalasin B and PBP10. (B) The figure shows the kinetics of the neutrophil response to WKYMVM in the absence (solid line) and presence of PBP10 (broken line). The results obtained are summarized in the inset and expressed as the integral values of oxidase activities from the controls (presence of cytochalasin B but not PBP10) and the activities in the presence of both cytochalasin B and PBP10.

To investigate the involvement of cytoskeleton in the PBP10-sensitive signal transduction pathway, neutrophils were treated with cytochalasin B prior to activation by a receptor agonist (in the presence or absence of PBP10). Interestingly, the receptor selectivity of PBP10's inhibitory effect was lost when the receptors were first uncoupled from the cytoskeleton by cytochalasin B (Fig. 5). In accordance with the findings reported above, the NADPH-oxidase activity induced by WKYMVM was largely inhibited by PBP10 also in the presence of cytochalasin B. However, the drug introduced a PBP10 sensitivity also in the FPR-induced response. The inhibition of the FPR-mediated response required higher concentrations of PBP10, and the response was only partly inhibited suggesting that the PBP10-insensitive as well as the PBP10-sensitive signaling pathways were triggered simultaneously during activation of FPR uncoupled from the cytoskeleton. The transient rise in intracellular calcium induced by the active receptors was not affected by cytochalasin B (data not shown). In conclusion, both FPRL1 and FPR appear to possess the ability to activate neutrophils via a signaling route that is sensitive to PBP10, but that this signaling pathway normally is blocked for FPR, through association of the receptor with the cytoskeleton.

## Discussion

The two formyl peptide receptor family members FPR and FPRL1 possess a high degree of amino acid identity in the signaling cytoplasmic domains [7], and the cell functions induced by the FPR and FPRL1 agonists are in most respects identical [10-15]. The indistinguishable responses of activated FPR and FPRL1 respectively, are expected, based on the fact that the two receptors are very similar in the regions suggested to be of importance for their interaction with the signaling, pertussis toxin-sensitive heterotrimeric G-protein. Dysfunctional variant FPR alleles (F110 replaced by an S and C126 replaced by a W) have been described that are associated with juvenile periodontitis and a deficiency in G-protein coupling [29,30], but FPRL1 contains the functional amino acids of FPR both in position 110 and in 126. Other G-protein coupling structures, identified through expression of different FPR mutants, suggest that the N-terminal part of the second transmembrane domain (S63 and D71) and the C-terminal interface of the third transmembrane domain (R123, C124 and C126) may be sites of interaction between the receptor and the G-protein [31]. In FPRL1 all but one of the amino acids in these suggested interaction sites are identical with those in FPR, and the signal-regulating NPXXY motif in the seven transmembrane domain (highly conserved among all GPCRs) is identical (sequence NPMLY [32]) in the two receptors. The exception is the serine in position 63 that in FPRL1 is replaced by a cysteine, but this difference seems to be of minor importance with respect to the effect of pertussis toxin, since both receptors are sensitive to the toxin.

Despite the indistinguishable activation by FPR and FPRL1, functional differences between these two highly homologous receptors emerge when they are challenged by the membrane permeant polyphosphoinositide-binding peptide PBP10 (rhodamine B-QRLFQVKGRR) prior to activation. The FPRL1-mediated neutrophil activity was totally inhibited by the peptide PBP10 whereas there was no effect on FPR-mediated responses. As mentioned, the amino acids in the PBP 10 peptide correspond to the PIP_2 _binding region segment 2 of the cytoskeletal protein gelsolin [16]. One of the enzymes competing with PBP10 for PIP_2 _would be the phosphoinositide remodeling enzyme PI3K that converts PIP_2 _into phosphatidylinositol 3,4,5-trisphosphate (PIP_3_) which is of importance for cell locomotion and the associated dynamic reorganization of cytoskeletal components. The precise target for PIP_3 _has however not yet been defined [33]. The inhibition of neutrophil function by Wortmannin, an inhibitor of PI3K, suggests that this signaling pathway is of importance for the NADPH-oxidase activity but the PI3K inhibitor lacked receptor specificity (i.e., both FPR and FPRL1 induced responses were inhibited) and we can thus rule out that the mechanism behind the PBP10 effect is a direct interference with the PI3K pathway.

The basic properties of PBP 10 have been described earlier [16,21,34], and it has been shown that when coupled to rhodamine B the peptide possesses not only PIP_2 _binding activity but also crosses the cell membrane of neutrophils and other cells. Interaction of rhodamine labeled peptides with PIP_2 _depends on the peptide sequence, and truncation of the peptide reduces the PIP_2 _binding affinity [21]. The truncated peptides were still membrane permeable and affected the oxidase activity only at higher concentrations. The unconjugated peptide QRLFQVKGRR exhibiting the same PIP_2_-binding characteristics as PBP10 but lacking the ability to cross the plasma membrane [16] had no effect on neutrophil oxidase activity. Taken together these data suggest that membrane permeation is required and PIP_2 _binding is of importance for peptide-induced inhibition.

Plasma membrane localized PIP_2 _is utilized for generation of IP_3 _which in turn is responsible for inducing transients in intracellular calcium [22]. Triggering of cells with fMLF or WKYMVM induced a rapid and transient increase in cytosolic calcium, but PBP10 did not have any inhibitory effect on the calcium transient. The Ca^2+ ^elevation has been claimed to be required but not sufficient for the generation of an NADPH-oxidase activating signal from FPR [23,24]. Likewise the mobilization of granule localized reserve pools of cell-surface receptors [25], has been suggested to be directly regulated by the cytosolic concentration of free Ca^2+ ^[26,27]. These experimental evidences rely, however, on methods that cannot distinguish a dependency on basal Ca^2+ ^levels from a requirement for a Ca^2+ ^transient, and we have earlier shown that receptor mobilization can occur and the oxidase can be activated without any transient rise in cytosolic Ca^2+ ^[13,35,36]. Despite the fact that PBP10 was without effect on the transient rise in intracellular Ca^2+ ^induced by WKYMVM, the secretory response was blocked and PBP10 selectively inhibited the FPRL1 induced granule mobilization.

The GPCR family is very diverse and the transmission of signals by such receptors is a critical function in many cell/organ systems. Signaling through GPCRs is highly complex, evidently not only with respect to the wide variety of mechanisms that regulate different functional responses [2], but also with respect to the pathways used to regulate a defined cellular response through closely related receptors. The two FPR and FPRL1 genes, although originating from a common ancestral gene, appear to have undergone markedly different evolutionary events [37]. In contrast to FPR, which is characterized by a relatively high degree of single nucleotide polymorphism (five non-synonymous and two synonymous identified [37]), no FPRL1 polymorphism has been found. Only one of the FPR polymorphisms is located in the cytoplasmic regions; and the variant (containing the A346 → E exchange) has the same amino acid in that position in the cytoplasmic tail as FPRL1. A direct comparison of the amino acid sequences of FPR and FPRL1 reveal very small differences between the receptors in all intracellular domains except for the C-terminal tail. In the first intracellular loop the H57 in FPR is replaced by an R (H57 → R) in FPRL1, in the second loop differences are found in V125 → I and T133 → A, and in the third loop the Q231 → K and L233 → M exchanges are found. The only major differences between the two receptors are found in the cytoplasmic C-terminal tail in which 13 out of 45 amino acids differ, and it is worth noting that the amino acid exchange in nine of the positions that differ between FPR and FPRL1 are potential targets for phosphorylation. Five potential phosphorylation sites, suggested to be of importance for arrestin-binding and receptor desensitization in FPR [38], are missing in FPRL1 while two new sites have been added in this receptor. It seems reasonable to assume that the signaling route that is sensitive to PBP10 originates from this region of the FPRL1 receptor. Despite the fact that the differences between FPR and FPRL1 are limited, the identification of putative sites in FPRL1 of importance for the PBP10 sensitivity cannot be achieved through experiments performed with receptor chimeras, site-directed receptor mutants or deletions, since no cells equipped with the required effector functions are available for expression of the receptors.

Regulation of FPR signaling has been suggested to involve mechanisms that depend on direct receptor interaction with the membrane cytoskeleton [28]. It is well known that the neutrophil response to FPR and FPRL1 agonists is both augmented and prolonged in the presence of cytochalasin B [15], a drug that inhibits re-organization of actin polymers and uncouples the receptors from the cytoskeleton [13,36]. The cytoskeleton is part of the signaling modulating machinery and we show that the receptor selectivity of the PBP10 inhibitory effect was lost when the receptors were uncoupled from the cytoskeleton. In accordance with the earlier described findings, the NADPH-oxidase activity induced by WKYMVM was largely inhibited by PBP10 also in the presence of cytochalasin B, but the drug introduced this sensitivity when fMLF was used as the triggering agent. The inhibition of the FPR-mediated response required higher concentrations of PBP10, and the response was only partly inhibited suggesting that the PBP10-insensitive as well as PBP10-sensitive signaling pathways were activated simultaneously by FPR's uncoupled from the cytoskeleton. It is interesting to note that when FPR was uncoupled from the cytoskeleton by cytochalasin B, PBP10 affected the sustained generation of superoxide but not the initial rate of production. This suggests that different signals are responsible for the triggering of the oxidase in the early and late phases of the response, respectively. It is reasonable to assume that the PBP10 sensitive signal generated by the uncoupled FPR is identical to that generated by FPRL1, however, we cannot at present exclude the possibility that also a novel FPR-triggered pathway is blocked by PBP10. The transient rise in intracellular calcium induced by the active receptors was not affected by cytochalasin B (data not shown), suggesting that this signaling route does not depend on interaction with the cytoskeleton. A possible explanation for the effects when PBP10 and cytochalasin B are combined is that signaling G-proteins compete with cytoskeletal proteins for the same site on FPR, and that this interaction involves a region of the receptor that differs between FPR and FPRL1. A 15 amino acid long sequence in FPR (^322^FPR^336^) has a fairly high (45–50%) identity with the actin-binding cytoskeletal proteins vinculin and coronin, and this region also participates in FPR interaction with the G-protein [28]. It is of interest to notice that the amino acid sequence in this region of FPRL1 differs from that of FPR in five positions and four of these are (in FPR) potential phosphorylation sites. Phagocytes express predominantly the G_i_α_2 _complex of the pertussis toxin sensitive G-proteins and, to a lesser extent G_i_α_3 _[39]. The molecular mechanism behind the difference in sensitivity to PBP10 between FPR and FPRL1 could possibly be that the receptors couple to different G-protein subtypes. FPR has, however, been shown to couple to both G-protein subtypes with similar efficiency [40]. It is however important to note that these experiments were performed in receptor-expressing cells in which nothing is known about the linkage between the receptors and the cytoskeleton and in which the proper cell function repertoire is missing.

The relation of the biochemical/biophysical activities of the PBP10 peptide to its effect on the FPRL1-triggered cell function is not obvious and is likely to be complex. The sensitivity to PBP10 seems to be unique to FPRL1, but this receptor is expressed also in other cells such as astrocytes, neuroblastoma, and microglia cells [8]. FPR is also expressed in other cell types and whether the receptor selectivity/specificity of PBP10 is maintained in other cells remains to be determined. The biochemical/biophysical characterization of the ten strategically organized basic and hydrophobic amino acids of the gelsolin molecule included in PBP10 reveal that it may interact with a broad range of negatively charged phosphomonoesters and hydrophobic acyl chains of anionic phospholipids [41,42]. This suggests that in addition to blocking/competing activities which involve proteins that are regulated by cellular phosphoinositides, the peptide may function as a buffer of bioactive and signaling lipids. Although elucidation of the step in signal transduction that is disrupted by PBP10 requires much additional work, the receptor-specific and signal-selective effects of this peptide on neutrophil functions suggest that it has a potential as a tool to manipulate and help define how GPCRs produce and integrate the signals generated from activated receptors and to probe new signaling functions of polyphosphoinositides as well as defining the role as promotor/blocker of G-protein signaling of different cytoskeletal proteins.

## Conclusions

The neutrophil formyl peptide receptor family members FPR and FPRL1 share 69% of amino acid identity and mediate almost indistinguishable cellular responses. Thus, the assumption that FPR and FPRL1 use the same signaling pathways has been generally accepted. However, in this study we clearly demonstrate a fundamental difference in intracellular signaling between these two very closely related neutrophil formyl peptide receptor members, one being PBP10 sensitive and the other not. This novel PBP10 sensitive signaling pathway utilized by FPRL1 is also used by FPR but only when the cytoskeleton network is disrupted.

## Methods

### Isolation of human neutrophils

Neutrophil granulocytes were isolated from buffy coats obtained from healthy adults. After dextran sedimentation at 1 × g, hypotonic lysis of the remaining erythrocytes, and centrifugation in a Ficoll-Paque gradient [43], the neutrophils were washed twice and resuspended (1 × 10^7 ^/ml) in Krebs-Ringer phosphate buffer containing glucose (10 mM), Ca^2+ ^(1 mM), and Mg^2+ ^(1.5 mM) (KRG; pH 7.3). The cells were stored on melting ice and used within 120 min of preparation.

### Chemoattractants and stimuli

The hexapeptide Trp-Lys-Tyr-Met-Val-Met-NH_2 _(WKYMVM) and the annexin I peptide (annexin_9–25 _Ac-QAWFIENEEQEYVQTVK) were synthesized and HPLC-purified by Alta Bioscience (University of Birmingham, Birmingham, United Kingdom), and Ross-Petersen ApS (Holte, Denmark). The formylated peptide N-formyl-Met-Leu-Phe (fMLF), C5a, and, phorbol myristate acetate (PMA) were from Sigma Chemical Co. (St. Louis, Missouri). IL-8 was from R&D systems (Minneapolis, MN) and dissolved in KRG containing 0.5% (w/v) bovine serum albumin. Serum amyloid A (SAA) was from Pepro Tech Inc. (UK). The C5a and SAA were dissolved in water while the other peptide agonists were dissolved in dimethyl sulfoxide to 10^-2 ^M and stored at -70°C until use. Further dilutions were made in KRG.

### PBP10 synthesis

The peptide QRLFQVKGRR (gelsolin residues 160–169) and related peptides were prepared by solid phase peptide synthesis and coupled to rhodamine as described earlier [16].

### Neutrophil NADPH-oxidase activity

Neutrophil production and release of superoxide anions was measured by means of an isoluminol-enhanced chemiluminescence (CL) assay [44]. The CL activity was measured in a six-channel Biolumat LB 9505 apparatus (Berthold Co., Wildbad, Germany), using disposable 4-ml polypropylene tubes with a 900-μl reaction mixture containing 10^5^–10^6 ^neutrophils, horseradish peroxidase (4 U) and isoluminol (20 μM) with and without cytochalasin B or PBP10. The measuring tubes were equilibrated for 5 to 10 minutes at 37°C and the cells were activated by addition of a receptor specific peptide agonist, fMLF for FPR and WKYMVM for FPRL1. The light emission was recorded continuously. Details about the CL technique are given in [45].

### Determination of changes in cytosolic calcium

Neutrophils at a density of 2 × 10^7^/ml in Ca^2+^-free KRG supplemented with bovine serum albumin (BSA, 0.1%) were incubated with the acetoxymethylated derivative fura-2/AM (2 μM) at room temperature for 30 minutes. The cells were washed twice and resuspended in KRG, adjusted to 2 × 10^7^/ml and kept protected from light on ice until use. Cells with or without PBP10 were equilibrated for 5 minutes at 37°C, after which the peptide agonist was added. The fura-2 fluorescence was followed with a luminescence spectrometer (LS50B; Perkin Elmer Corp.) using excitation wavelengths of 340 nm and 380 nm, and an emission wavelength of 510 nm and the [Ca^2+^]_i _was calculated as described earlier [46].

### Determination of receptor exposure by FACS analysis

The exposure of CR3 (CD11b/CD18) on the neutrophil cell surface was assessed by immunostaining and FACS-analysis. Cells challenged with peptide agonists with or without PBP10 were fixed with ice-cold paraformaldehyde and washed with FACSwash (PBS, 0.02% NaN_3_), after which the cells were incubated at 4°C with phycoerythrin-conjugated anti-CR3 antibodies (CD11b; Becton Dickinson 10 μl/10^6 ^cells) before analysis using a FACScan (Becton Dickinson, Mountain View, CA).

### Reagents

Horseradish peroxidase (HRP) was from Boehringer-Mannheim (Mannheim, Germany). Isoluminol, cytochalasin B, and pertussis toxin were purchased from Sigma. Dextran and Ficoll-Paque were from Pharmacia (Uppsala, Sweden), and Wortmannin was from Calbiochem (La Jolla, CA). Fura-2AM was from Molecular Probes Inc. (Eugene, OR).

### Statistic analysis

Two-tailed, paired Students's t-tests were performed to determine statistical significance, and a P-value of < 0.05 was regarded as significant.

## Abbreviations

GPCR – G-protein coupled receptor

PBP – Phosphoinositide binding peptide

FPR – formyl peptide receptor

FPRL1 – formyl peptide receptor-like 1

PIP2 – phosphatidylinositol 4,5-bisphosphate

PI3K – phosphatidylinositol 3-kinase

fMLF – formyl-methionly-leucyl-phenylalanin

## Authors' contributions

The scientific question raised in the paper was formulated during discussions between HF and CD, about the mechanisms behind receptor activation/deactivation/reactivation (see ref [38]). HF was responsible for most of the experiments but LB, JK and CM performed some of the experiments using techniques developed by CD and AK. PJ provided the PIP-binding peptides as well as suggestions for experiments. HF and CD wrote the first version of the paper, but contributions from all authors were important for the final outcome of the paper.
